# Early Initiation of Smoking and Alcohol Drinking as a Predictor of Lower Forearm Bone Mineral Density in Late Adolescence: A Cohort Study in Girls

**DOI:** 10.1371/journal.pone.0046940

**Published:** 2012-10-18

**Authors:** Raquel Lucas, Sílvia Fraga, Elisabete Ramos, Henrique Barros

**Affiliations:** 1 Department of Clinical Epidemiology, Predictive Medicine and Public Health, University of Porto Medical School, Porto, Portugal; 2 Institute of Public Health of the University of Porto, Porto, Portugal; University of Southampton, United Kingdom

## Abstract

**Background:**

Adolescence is a critical stage for bone accrual. It is also decisive for the establishment of behaviors such as smoking and alcohol drinking.

**Objective:**

To quantify the short- and long-term associations between smoking and drinking initiation and bone mineral density in adolescent girls.

**Methods:**

We used prospective data from 731 girls identified in public and private schools in Porto, Portugal. Evaluations were conducted when participants were 13 and 17 years old. Bone mineral density (BMD) was measured at the forearm by dual-energy X-ray absorptiometry and weight, height and fat-free mass were measured. Pubertal development status was estimated using menarche age. Self-administered questionnaires were used to collect data on smoking and alcohol drinking, physical exercise and calcium and vitamin D intakes. BMD in early and late adolescence was analyzed as a continuous or dichotomous (Z-score cutoff: −1.0) variable. Associations were calculated using linear or logistic regression.

**Results:**

Over one quarter of these girls had tried smoking by 13, while 59% had drunk alcoholic beverages and 20% had experienced both behaviors by that age. Lower mean BMD at 17 years of age was observed in girls who had ever smoked by 13, as well as in those who reported drinking at that age. There were no significant cross-sectional associations between experience and frequency of smoking or drinking and BMD at 13 years of age. However, we observed significant associations between BMD z-score<−1 in late adolescence and having ever smoked by 13, after adjustment for menarche age and sports practice, (OR = 1.92; 95% CI: 1.21, 3.05) and with ever smoking and drinking in the same period (OR = 2.33; 95% CI: 1.36, 4.00).

**Conclusion:**

Our study adds prospective evidence to the role of early initiation of smoking and alcohol drinking as relevant markers of lower bone mineral density in late adolescence.

## Introduction

It is currently believed that bone quality is characterized by important tracking throughout the life course, in such a way that the probability of fragility fracture in old age may be partly traced back to the bone properties attained during the first decades of life [1]. Peak bone strength, through its surrogate peak bone mass, was proposed as a potential determinant of fragility fractures in adulthood, with even larger influence than the rate of bone loss [2]. As a consequence, increasing interest has been devoted to the study of bone mineralization in childhood and adolescence and particularly to the factors that interfere with this process [3].

Family studies have shown that most variability in bone properties observed within populations is hereditary [4]. However, known genetic polymorphisms explain only a small fraction of this variation and complex biological interactions preclude a clear distinction of the phenotypic effects of common genetic makeup from those of shared environments [5]. Regardless of this intrinsic challenge, environmental factors are thought to act essentially by modulating the achievement of an individual's full genetic potential for bone mass [6]. Therefore, in the primary prevention of fragility fractures in old age, modifiable determinants of bone properties early in life are of evident interest.

Causal roles for smoking and alcohol intake in the pathogenesis of fragility fractures during adulthood have long been suggested. Presently, substantial epidemiological evidence has accumulated of higher fracture rates among smokers [7] and heavy drinkers [8]. Although there are several possible biological mechanisms underlying such relations whose relative importance remains to be clarified, bone properties seem to be major mediators of such pathways [9]–[11].

Most research on the effects of smoking and alcohol on bone health has targeted adulthood, when the major concerns are rate of bone loss and trauma severity. In fact, even though childhood and adolescence are the periods of highest bone mineral accrual, critical for peak bone mass attainment, there is comparative scarcity of research on the association between those behaviors and bone parameters in the first decades of life. Studies that examined those relations early in life yielded heterogeneous results, some proposing absent or weak effects and others describing inverse associations since young ages. However, most evidence was cross-sectional [12], [13], from small samples of specific populations [14]–[16] or targeting wide age ranges [17]–[20].

Adolescence is a critical stage not only for bone accrual but also for the establishment of potentially deleterious health-related behaviors [21]. Early uptake of smoking and drinking can be viewed simultaneously as a component as well as marker of a set of such unhealthy exposures. Thus, it is relevant to assess whether initiation of these behaviors in the general adolescent population may predict suboptimal bone properties in the short- and long-terms.

Therefore, by prospectively evaluating a population-based cohort of girls, we aimed at quantifying the associations between early initiation of smoking and alcohol drinking and forearm bone mineral density in early and late adolescence.

## Methods

In the present study, we used prospective data collected from 731 adolescent girls recruited and followed up as part of the Epidemiological Health Investigation of Teenagers in Porto (EPITeen), a cohort of urban adolescents born in 1990.

### Cohort recruitment (13 years old) and follow-up evaluation (17 years old)

During the 2003/2004 school year, the research team approached all public and private schools in Porto, Portugal, that provided teaching to children born in 1990. Forty-six out of 51 eligible schools agreed to participate by facilitating the contact between researchers and students and their families. The aims and procedures involved in the study were explained to parents, teachers, and children through meetings arranged in each school as well as through written materials. In participant schools, we identified 2787 eligible boys and girls of whom 78% agreed to participate and provided information for at least part of the protocol. The recruitment process yielded a baseline sample of 1116 13-year-old girls. Recruitment procedures have been described in detail elsewhere [22].

The first follow-up evaluation of this cohort was conducted during the 2007/2008 school year. Re-evaluations were scheduled by contacting schools or participants directly, using contact details provided during the recruitment period. Of the 1116 girls initially recruited, 892 were successfully reevaluated at 17 years of age (attrition rate: 20.1%).

### Ethics Statement

Written informed consent was obtained from all participants and their legal guardians in each evaluation. The study protocol was defined according to the Declaration of Helsinki and was approved by the Ethics Committee of the University Hospital of São João in Porto, Portugal.

### Physical examination

The same protocol for physical examination was used in baseline and follow-up evaluations. Bone mineral density (BMD) was measured in g/cm^2^ at the distal radius of the non-dominant forearm by dual-energy X-ray absorptiometry (DXA) using a GE Lunar® Peripheral Instantaneous X-ray Imager. In case of reported previous fracture of the non-dominant arm, the dominant arm was the one assessed. Anthropometry was obtained while the adolescent stood barefoot in light indoor clothing. Weight was measured to the nearest tenth of kilogram (Tanita®), and height was measured in centimeters, to the nearest tenth, using a portable stadiometer (Seca®). Fat-free mass was estimated to the nearest tenth of kilogram using bioelectrical impedance (Tanita® TBF-300). Pubertal development status was measured using menarche age in years, obtained through a self-administered questionnaire.

### Behavioral variables

Behavioral characteristics were collected at 13 and 17 years of age, using self-administered questionnaires. One of the questionnaires was completed at home with parental assistance and included issues such as dietary intake, vitamin or mineral supplements, physical activity, and parental education (measured as the number of schooling years completed by the parent with the highest formal education). Sensitive behavioral topics (smoking, alcohol drinking and oral contraceptive use) were inquired trough a questionnaire filled in at school without parental assistance and ensuring privacy.

Smoking behavior was assessed at each age using the question “Have you ever smoked?”. Adolescents were classified as never smokers if they reported having never tried smoking in both evaluations, as ever smokers between 13 and 17 years of age if they reported having never smoked in the 13-year-old questionnaire but having smoked in the 17-year-old questionnaire and as ever smokers at 13 if they reported having ever tried smoking in the 13-year-old questionnaire. At each age, among ever smokers, the frequency of smoking was classified using the following categories: has tried but does not currently smoke, smokes but not every day, smokes at least once a day.

Drinking behavior was assessed through the question “Have you ever drunk an alcoholic beverage?”. Adolescents were classified as never drinkers if they had never tried drinking in both evaluations, as ever drinkers between 13 and 17 years of age if they reported not drinking in the 13-year-old questionnaire but drinking in the 17-year-old questionnaire and as drinkers at 13 if they reported drinking in the 13-year-old questionnaire. At each age ever drinkers were categorized in one of the following groups: has tried but does not currently drink, drinks less than once a week, drinks at least once a week but not every day, drinks every day.

Physical exercise practice was assessed at 13 and 17 years of age using the question “Besides school time, how frequently do you practice sports for at least 20 minutes?”. Participants were grouped into one of two categories: those practicing sports up to once weekly and those who practiced two or more times per week.

In order to assess smoking and drinking as markers of a set of adverse health behaviors, we used previously-defined behavioral clusters as exposures. These groups were identified in this population using the natural structure of the following set of data: sports activities, fruit intake, sleeping hours and time spent in sedentary activities, as well as tobacco and alcohol use [23]. Adolescents were classified in one of three following groups according to behavioral aggregation: healthiest (cluster 1), intermediate (cluster 2) and least healthy (cluster 3).

Dietary habits in the 12 months preceding the evaluation were assessed at 13 years of age using a food frequency questionnaire that was designed by adapting to the adolescent population a questionnaire previously validated in Portuguese adults [24]. The questionnaire comprised 92 food items, whose average frequency of consumption was asked to participants. This information, together with a previously defined average portion size, was used to compute the average daily intake of each nutrient using Food Processor Plus®. Using this procedure, we estimated daily intakes of calcium (mg/day) and vitamin D (µg/day).

Adolescents were also asked about vitamin or mineral supplementation in the previous 12 months, from which we extracted information about the use of specific calcium and/or vitamin D supplements (Anatomical Therapeutic Chemical code A12A). Additionally, current oral contraceptive use was obtained by combining information from reported medication in the previous month with a specific question inquiring about current use of the birth control pill.

### Data analysis

The main outcome, bone mineral density, was used either as a continuous variable or as z-scores, obtained by calculating the difference between each BMD value and the sample mean, divided by the sample standard-deviation. The clinical cutoff for the diagnosis of low bone mass for chronologic age is a sex- and age-specific z-score under −2.0 [25]. Since this is a population based sample, we found a low frequency of this pathological change (1.5% prevalence at 13 and 2.0% at 17 years of age). Nevertheless, we aimed to characterize those individuals that, within the normal range, had lower bone density. Since there are no agreed cutoffs for bone mineral density in the general non-osteoporotic pediatric population, we opted for using an *a priori* defined cutoff based on z-score units (widely considered the measure with the clearest clinical significance): adolescents were classified as having high or low BMD at each age, according to whether their z-score was above or below −1.0 (this cutoff corresponded to BMD values of 0.303 g/cm^2^ at 13 and 0.386 g/cm^2^ at 17 years of age). To assess possible confounders or mediators, menarche age, anthropometric parameters, nutrient and supplement intake, physical activity, oral contraceptives, parental education, and behavioral clusters were described according to classes of smoking and drinking behaviors, as well as according to bone mineral density z-score classes (≥−1SD or <−1 SD) at 13 and 17 years of age. One-way ANOVA was used to compare means between groups. Proportions were compared using Chi-square of Fisher's exact tests, as appropriate. We calculated adjusted mean BMD values and 95% confidence intervals (95% CI) using linear regression. Logistic regression was also used to estimate the magnitude of the adjusted associations (odds ratios and 95% CI) of smoking and drinking precocity with low bone mineral density in late adolescence.

From a total of 892 girls assessed both at 13 and 17 years of age, 731 had complete information regarding smoking and drinking habits and forearm bone densitometry. Missing data are mostly due to the fact that the bone density equipment was not available during a short period of time. By comparing the 385 girls in the initial cohort who were not analyzed with the remaining 731, there were no differences in the frequency of ever smoking at 13 (26.6% vs. 26.8%, respectively, p = 0.933) or in the proportion post-menarcheal at recruitment (84.3% vs. 86.3%, respectively, p = 0.404). However, girls who were not included in the present analysis reported less frequently having ever drunk an alcoholic beverage at 13 years of age (47.2% vs. 58.4%, p = 0.001), and had higher mean baseline BMD (0.368 vs. 0.358 g/cm^2^, p = 0.027).

## Results

### Forearm bone mineral density

In this sample of 731 girls, mean (SD) forearm bone mineral density was 0.358 (0.057) at 13 and 0.434 (0.052) g/cm^2^ at 17 years of age. Among the 125 girls who had low bone mineral density (<−1 SD below the mean) at 13 years of age, 56% remained in the low BMD group at 17. Of the 606 without low BMD at 13, 90% remained in that category at 17 years of age. Low BMD in both ages was more frequent in girls with later menarche, lower body mass index and lower fat-free mass. Although non-significantly, the proportion of girls reporting regular sports practice at 17 years of age was lower among those with BMD z-score<−1 at the same age, but no clear association was found at 13 years of age. No association was found between BMD and calcium and vitamin D dietary intakes or supplements, use of oral contraceptives, period of schooling of the most educated parent, or behavioral cluster (Table 1).

**Table 1:** Distribution of anthropometric and behavioral characteristics at 13 and 17 years of age according to bone mineral density (BMD) z-scores at 13 and 17 years of age.

	BMD at 13 years of age			BMD at 17 years of age		
	z-score≥−1†	z-score<−1	*p*	z-score≥−1†	z-score<−1	*p*
N (%)	606 (82.9)	125 (17.1)		599 (81.9)	132 (18.1)	
Mean (SD) menarche age (years)	12.1 (1.3)	13.2 (1.1)	*<0.001*	12.2 (1.3)	12.8 (1.3)	*<0.001*
Mean (SD) height at 13 (cm)	168.6 (6.2)	155.2 (7.8)	*<0.001*	158.4(6.4)	156.3 (7.0)	*<0.001*
Mean (SD) height at 17 (cm)	161.1 (6.1)	160.6 (7.0)	*0.392*	161.1 (6.3)	160.6 (6.0)	*0.440*
Mean (SD) weight at 13 (kg)	54.0 (9.4)	44.5 (7.5)	*<0.001*	53.5 (9.5)	47.0 (9.2)	*<0.001*
Mean (SD) weight at 17 (kg)	58.0 (9.2)	51.7 (7.3)	*<0.001*	58.1 (9.1)	51.9 (7.9)	*<0.001*
Mean (SD) body mass index at 13 (kg/m^2^)	21.4 (3.3)	18.4 (2.4)	*<0.001*	21.3 (3.3)	19.1 (3.1)	*<0.001*
Mean (SD) body mass index at 17 (kg/m^2^)	22.2 (3.7)	20.0 (2.3)	*<0.001*	22.2 (3.5)	19.9 (3.2)	*<0.001*
Mean (SD) fat-free mass at 13 (kg)	38.6 (3.9)	35.4 (3.9)	*<0.001*	38.6 (3.9)	35.8 (4.1)	*<0.001*
Mean (SD) fat-free mass at 17 (kg)	42.8 (3.7)	40.4 (3.7)	*<0.001*	42.9 (3.7)	40.3 (3.6)	*<0.001*
Mean (SD) calcium intake at 13 (mg/day)	1146.6 (464.0)	1127.5 (444.6)	*0.707*	1157.6 (467.4)	1073.7 (420.6)	*0.095*
Mean (SD) vitamin D intake at 13 (µg/day)	4.5 (2.6)	4.5 (2.4)	*0.881*	4.6 (2.6)	4.2 (2.4)	*0.183*
Number (%) using calcium/vit D supplements at 13	3 (0.5)	1 (0.8)	*0.528*	4 (0.7)	0 (0.0)	*>0.999*
Number (%) using calcium/vit D supplements at 17	‡	‡	‡	3 (0.5)	1 (0.8)	*0.550*
Number (%) reporting oral contraceptive at 17	‡	‡	‡	241 (40.2)	47 (35.6)	*0.376*
Number (%) practicing sports ≥2 times/week at 13	265 (44.5)	56 (47.1)	*0.603*	269 (45.8)	52 (40.6)	*0.284*
Number (%) practicing sports ≥2 times/week at 17	‡	‡	‡	252 (43.7)	45 (35.2)	*0.077*
Mean (SD) parental education (schooling years)	10.7 (4.4)	10.5 (5.0)	*0.644*	10.6 (4.4)	11.1 (4.9)	*0.234*
Number (%) in behavioral clusters*			*0.797*			*0.651*
Cluster 1	210 (39.9)	43 (43.5)		215 (41.5)	40 (36.6)	
Cluster 2	301 (57.1)	53 (53.5)		288 (55.7)	66 (60.6)	
Cluster 3	16 (3.0)	3 (3.0)		16 (3.1)	3 (2.8)	

P-values were calculated using chi-square of Fisher's exact tests, as appropriate (for the comparisons between proportions), or using Student's t-test (for the comparisons of means).

* Behavioral clusters defined using the following variables: sports activities, fruit intake, sleeping hours, time spent in sedentary activities, tobacco and alcohol use [23]. Adolescents were classified in one of three following groups according to behavioral aggregation: healthiest (cluster 1), intermediate (cluster 2) and least healthy (cluster 3).

† Z-score −1.0 cutoff corresponded to 0.303 g/cm^2^ at 13 and 0.386 g/cm^2^ at 17 years of age.

‡ Estimates are not presented because exposures are behaviors which took place in late adolescence while the outcome (BMD) refers to early adolescence.

### Smoking and alcohol drinking

Of the 731 girls evaluated, 712 and 716 provided information regarding ever smoking and drinking alcohol, respectively, in both evaluations.

At 13 years of age, over one quarter of the girls reported having ever smoked. Among ever smokers at 13, 189 provided useful frequency data, of which 165 (85.9%) reported not smoking regularly, 15 (7.8%) smoked but not every day while 9 (4.7%) smoked every day. Also about one quarter of girls had first tried smoking between 13 and 17 years of age and half remained never smokers up to 17 years of age. Among 329 girls who had ever smoked by 17 years of age and provided frequency data, 224 (66.9%) did not smoke regularly, 33 (9.8%) smoked but not every day and 72 (21.5%) reported smoking at least once a day.

Almost 60% of girls reported having ever drunk an alcoholic beverage by 13 years of age. Among the 414 ever drinkers at 13 who provided frequency information, 380 (91.8%) reported not drinking regularly, 26 (6.3%) drank under once a week and 8 (1.9%) reported drinking at least once a week. By 17 years of age, an additional 30% of girls had first tried drinking. Among 593 ever drinkers at 17 that provided frequency information, 273 (46.0%) reported not drinking regularly, 266 (44.8%) drank less than once a week and 54 (9.1%) drank at least once a week. Regarding both behaviors combined, one fifth of the sample reported having tried smoking and drinking by 13 years of age.

The characteristics of adolescents according to smoking and drinking behaviors are summed up in Table 2. Early initiation of both behaviors was more frequent among girls with earlier menarche: 12.0 was the mean menarche age among ever smokers and drinkers vs. 12.4 in the remaining girls. No differences in mean body mass index (BMI) or fat-free mass at 13 years of age were found between classes of smoking and drinking experience. However, an overall decreasing trend in weight, BMI and fat-free mass at 17 years of age with increasing smoking and drinking precocity was observed. Oral contraceptive use by 17 was more frequent in girls who tried smoking and drinking earlier in life. Decreasing trends across classes of smoking were found for mean calcium (p-value for linear trend: 0.149) and vitamin D (p-value for linear trend: 0.036) intakes. The frequency of reported regular sports practice in both evaluations decreased non-significantly with increasing smoking precocity but increased (significantly at 13 years of age) with drinking precocity. When compared to girls who had not tried smoking or drinking before 13 years of age and to those who reported one of those behaviors, an intermediate frequency of regular sports practice was found in girls who had tried both smoking and drinking by 13. Average parental formal education was highest among adolescents who reported drinking before 13 years of age, and lowest in those who had never tried drinking by 17 years of age.

**Table 2:** Distribution of anthropometric and behavioral characteristics at 13 and 17 years of age according to smoking and drinking categories.

	Never smoked at 17	Tried smoking after 13 but before 17	Tried smoking before 13	*p*	Never drank at 17	Tried drinking after 13 but before 17	Tried drinking before 13	*p*	Tried none before 13	Tried smoking or drinking before 13	Tried smoking and drinking before 13	*p*
n (%)	353 (49.6)	167 (23.5)	192 (27.0)		84 (11.7)	213 (29.8)	419 (58.5)		248 (35.4)	312 (44.6)	140 (20.0)	
Mean (SD) menarche age (years)	12.4 (1.3)	12.4 (1.2)	12.1 (1.3)	*0.024*	12.2 (1.5)	12.5 (1.2)	12.2 (1.3)	*0.082*	12.4 (1.3)	12.4 (1.3)	11.9 (1.3)	*0.003*
Mean (SD) height at 13 (cm)	157.8 (6.9)	158.8 (6.4)	158.2 (6.2)	*0.242*	156.6 (7.4)	157.7 (7.0)	158.5 (6.2)	*0.037*	158.0 (6.7)	158.5 (6.2)	157.3 (5.0)	*0.352*
Mean (SD) height at 17 (cm)	160.9 (6.4)	161.8 (6.0)	160.8 (6.1)	*0.231*	159.7 (6.5)	160.9 (6.5)	161.2 (6.3)	*0.126*	161.1 (6.3)	160.9 (6.2)	160.7 (5.8)	*0.818*
Mean (SD) weight at 13 (kg)	52.4 (10.4)	52.6 (9.8)	52.3 (8.5)	*0.969*	52.5 (11.2)	51.9 (10.2)	52.5 (9.2)	*0.707*	52.0 (10.5)	52.9 (10.0)	52.3 (7.9)	*0.540*
Mean (SD) weight at 17 (kg)	57.4 (9.7)	57.3 (9.3)	56.1 (8.4)	*0.265*	58.3 (10.3)	56.8 (9.5)	56.7 (8.9)	*0.338*	57.2 (9.8)	57.5 (9.4)	55.8 (8.0)	*0.188*
Mean (SD) body mass index at 13 (kg/m^2^)	20.9 (3.5)	20.8 (3.4)	20.9 (3.1)	*0.871*	21.3 (3.8)	20.8 (3.4)	20.9 (3.2)	*0.455*	20.8 (3.4)	21.0 (3.6)	20.7 (2.8)	*0.645*
Mean (SD) body mass index at 17 (kg/m^2^)	22.1 (3.3)	21.7 (3.7)	21.6 (3.4)	*0.145*	22.9 (3.7)	21.7 (3.8)	21.8 (3.1)	*0.020*	22.0 (3.6)	22.0 (3.6)	21.5 (2.8)	*0.301*
Mean (SD) fat-free mass at 13 (kg)	38.1 (4.3)	38.4 (3.9)	38.0 (3.8)	*0.588*	38.0 (4.6)	37.8 (4.4)	38.3 (3.8)	*0.463*	37.9 (4.4)	38.5 (4.0)	37.9 (3.6)	*0.166*
Mean (SD) fat-free mass at 17 (kg)	42.7 (4.1)	42.6 (3.5)	41.9 (3.6)	*0.054*	43.0 (4.1)	42.4 (4.0)	42.3 (3.7)	*0.312*	42.6 (4.0)	42.6 (3.8)	41.7 (3.5)	*0.042*
Mean (SD) calcium intake at 13 (mg/day)	1170.6 (461.2)	1158.7 (475.6)	1083.8 (447.3)	*0.149*	1140.6 (450.7)	1160.7 (453.4)	1133.3 (466.6)	*0.815*	1169.8 (440.8)	1155.2 (487.6)	1084.5 (422.2)	*0.251*
Mean (SD) vitamin D intake at 13 (µg/day)	4.7 (2.6)	4.4 (2.6)	4.2 (2.3)	*0.110*	4.6 (2.8)	4.8 (2.7)	4.4 (2.4)	*0.211*	4.8 (2.8)	4.5 (2.4)	4.2 (2.4)	*0.094*
Number (%) using calcium/vitamin D supplements at 13	0 (0.0)	2 (2.1)	2 (1.0)	*0.064*	1 (1.2)	1 (0.5)	2 (0.5)	*0.579*	1 (0.4)	2 (0.6)	1 (0.7)	*>0.999*
Number (%) using calcium/vitamin D supplements at 17	0 (0.0)	1 (0.6)	3 (1.6)	*0.040*	1 (1.2)	0 (0.0)	3 (0.7)	*0.318*	0 (0.0)	2 (0.6)	2 (1.4)	*0.194*
Number (%) reporting oral contraceptive at 17	97 (27.5)	82 (49.1)	102 (53.1)	*<0.001*	18 (21.4)	87 (40.8)	177 (42.2)	*0.002*	80 (32.6)	121 (38.8)	74 (52.9)	*<0.001*
Number (%) practicing sports ≥2 times/week at 13	167 (48.3)	73 (43.7)	79 (41.8)	*0.313*	32 (39.5)	95 (45.0)	191 (46.1)	*0.548*	108 (44.3)	150 (48.7)	57 (41.3)	*0.302*
Number (%) practicing sports ≥2 times/week at 17	155 (44.8)	66 (40.5)	72 (39.3)	*0.415*	24 (30.4)	82 (39.6)	187 (46.0)	*0.024*	90 (37.5)	145 (47.2)	54 (40.3)	*0.063*
Mean (SD) parental education (schooling years)	10.7 (4.5)	10.4 (4.6)	11.1 (4.5)	*0.344*	8.6 (4.0)	10.6 (4.5)	11.2 (4.5)	*<0.001*	10.1 (4.4)	11.0 (4.6)	11.5 (4.4)	*0.010*
Number (%) in behavioral clusters*				*<0.001*				*<0.001*				*<0.001*
Cluster 1	163 (51.6)	56 (37.6)	33 (20.8)		69 (97.2)	183 (98.9)	0 (0.0)		219 (100.0)	33 (11.7)	0 (0.0)	
Cluster 2	153 (48.4)	93 (62.4)	107 (67.3)		0 (0.0)	0 (0.0)	354 (95.9)		0 (0.0)	246 (86.9)	107 (87.7)	
Cluster 3	0 (0.0)	0 (0.0)	19 (12.0)		2 (2.8)	2 (1.1)	15 (4.1)		0 (0.0)	4 (1.4)	15 (12.3)	

P-values were calculated using chi-square of Fisher's exact tests, as appropriate (for the comparisons between proportions), or using one-way ANOVA (for the comparisons of means).

* Behavioral clusters defined using the following variables: sports activities, fruit intake, sleeping hours, time spent in sedentary activities, tobacco and alcohol use [23]. Adolescents were classified in one of three following groups according to behavioral aggregation: healthiest (cluster 1), intermediate (cluster 2) and least healthy (cluster 3).

### Smoking and/or drinking and 13-year-old bone mineral density

Table 3 presents mean BMD values at 13 years of age, according to smoking and alcohol drinking experience. Estimates are presented adjusted for menarche age and sports practice, and additionally adjusted for body mass index. Overall, we found no clear cross-sectional associations between BMD at 13 and smoking or drinking experience or frequency at the same age.

**Table 3:** Mean forearm bone mineral density (BMD) and 95% confidence intervals (95% CI) at 13 years of age according to smoking and drinking categories and adverse behaviors clusters.

		Mean (95% CI) forearm BMD at 13 years of age	
	n	Adjusted for menarche age and regular sports	Adjusted for menarche age, regular sports and body mass index
Smoking frequency at 13			
Never tried smoking	523	0.359 (0.355, 0.363)	0.357 (0.353, 0.362)
Tried but does not currently smoke	165	0.355 (0.347, 0.363)	0.361 (0.355, 0.368)
Smokes but not every day/Smokes at least once a day	24	0.347 (0.326, 0.367)	0.355 (0.349, 0.361)
p		0.366	0.410
Drinking frequency at 13			
Never tried drinking	298	0.359 (0.353, 0.365)	0.358 (0.353, 0.363)
Tried but does not currently drink	380	0.359 (0.354, 0.364)	0.359 (0.354, 0.363)
Drinks less than once a week	26	0.345 (0.325, 0.365)	0.351 (0.333, 0.368)
Drinks at least once a week but not every day/Drinks every day	8	0.353 (0.318, 0.388)	0.354 (0.323, 0.384)
p		0.590	0.826
Ever smoking or drinking before 13			
Tried none	248	0.358 (0.351, 0.364)	0.358 (0.352, 0.363)
Tried one	312	0.361 (0.356, 0.367)	0.361 (0.356, 0.366)
Tried both	140	0.351 (0.343, 0.360)	0.353 (0.346, 0.361)
p		0.146	0.248
Behavioral cluster* at 13 years of age			
Cluster 1	253	0.359 (0.353, 0.365)	0.358 (0.353, 0.364)
Cluster 2	354	0.359 (0.354, 0.364)	0.359 (0.355, 0.364)
Cluster 3	19	0.346 (0.323, 0.368)	0.350 (0.331, 0.370)
p		0.528	0.697

P-values were calculated using one-way ANOVA.

* Behavioral clusters defined using the following variables: sports activities, fruit intake, sleeping hours, time spent in sedentary activities, tobacco and alcohol use [23]. Adolescents were classified in one of three following groups according to behavioral aggregation: healthiest (cluster 1), intermediate (cluster 2) and least healthy (cluster 3).

### Smoking and/or drinking and 17-year-old bone mineral density

In Table 4, average BMD is presented according to experience and frequency of smoking and drinking in early and late adolescence. After adjustment for menarche age and sports practice, forearm BMD at 17 years of age was lower among girls who ever tried smoking before 13 years of age (0.426 vs. 0.437 g/cm^2^ among never smokers), as well as in those who had ever drunk an alcoholic beverage before 13 (0.431 vs. 0.447 g/cm^2^ among never drinkers). The significance of both associations was attenuated after adjustment for body mass index. No significant associations were found between smoking or drinking frequency and 17 year-old BMD, but there was a decreasing trend of BMD with increasing drinking frequency at 13 (adjusted BMD was 0.439 in those who never drank vs. 0.414 g/cm^2^ in those who drank at least once a week) and at 17 years of age (adjusted BMD was 0.438 in those who never drank vs. 0.420 g/cm^2^ in those who drank daily). BMD in late adolescence was significantly lower in girls who had tried smoking and drinking before 13 (0.422 vs. 0.438 g/cm^2^ in those who had tried none by that age).

**Table 4:** Mean forearm bone mineral density (BMD) and 95% confidence intervals (95% CI) at 17 years of age according to smoking and drinking categories and adverse behaviors clusters.

		Mean (95% CI) forearm BMD at 17 years of age	
	N	Adjusted for menarche age and regular sports	Adjusted for menarche age, regular sports and body mass index
Smoking frequency at 13			
Never tried	523	0.437 (0.433, 0.442)	0.436 (0.432, 0.440)
Tried but does not currently smoke	165	0.425 (0.417, 0.433)	0.426 (0.419, 0.434)
Smokes but not every day/Smokes at least once a day	24	0.431 (0.410, 0.452)	0.433 (0.413, 0.452)
p		0.217	0.068
Smoking frequency at 17			
Never tried	390	0.436 (0.431, 0.441)	0.435 (0.430, 0.440)
Tried but does not currently smoke	224	0.435 (0.428, 0.442)	0.436 (0.429, 0.442)
Smokes but not every day	33	0.418 (0.400, 0.436)	0.421 (0.404, 0.438)
Smokes at least once a day	72	0.429 (0.417, 0.441)	0.432 (0.420, 0.443)
p		0.257	0.438
Ever smoking in adolescence			
Never smoked at17	353	0.437(0.432, 0.443)	0.436 (0.431, 0.441)
Tried smoking after 13 but before 17	167	0.438 (0.430, 0.445)	0.438 (0.431, 0.445)
Tried smoking before 13 years of age	192	0.426 (0.418, 0.433)	0.428 (0.421, 0.435)
p		0.030	0.103
Drinking frequency at 13			
Never tried drinking	298	0.439 (0.434, 0.445)	0.438 (0.433, 0.444)
Tried but does not currently drink	380	0.433 (0.428, 0.438)	0.433 (0.428, 0.438)
Drinks less than once a week	26	0.420 (0.400, 0.439)	0.428 (0.410, 0.447)
Drinks at least once a week but not every day/Drinks every day	8	0.414 (0.378, 0.449)	0.417 (0.384, 0.449)
p		0.084	0.282
Drinking frequency at 17			
Never tried drinking	120	0.438 (0.429, 0.448)	0.434 (0.425, 0.442)
Tried but does not currently drink	273	0.436 (0.430, 0.442)	0.436 (0.430, 0.442)
Drinks less than once a week	266	0.432 (0.426, 0.438)	0.434 (0.428, 0.439)
Drinks at least once a week but not every day/Drinks every day	54	0.420 (0.406, 0.434)	0.424 (0.411, 0.438)
p		0.157	0.471
Ever drinking in adolescence			
Never drank at17	84	0.447 (0.435, 0.458)	0.441 (0.430, 0.451)
Tried drinking after 13 but before 17	213	0.436 (0.430, 0.443)	0.437 (0.431, 0.444)
Tried drinking before 13 years of age	419	0.431 (0.426, 0.436)	0.432 (0.428, 0.437)
p		0.045	0.216
Ever smoking or drinking before 13			
Tried none	248	0.438 (0.431, 0.444)	0.438 (0.432, 0.444)
Tried one	312	0.437 (0.431, 0.443)	0.436 (0.431, 0.442)
Tried both	140	0.422 (0.413, 0.430)	0.424 (0.416, 0.433)
p		0.006	0.023
Behavioral cluster* at 13 years of age			
Cluster 1	253	0.439 (0.433, 0.446)	0.438 (0.432, 0.444)
Cluster 2	354	0.432 (0.427, 0.438)	0.433 (0.428, 0.438)
Cluster 3	19	0.436 (0.412, 0.459)	0.439 (0.417, 0.461)
p		0.307	0.399

P-values were calculated using one-way ANOVA.

* Behavioral clusters defined using the following variables: sports activities, fruit intake, sleeping hours, time spent in sedentary activities, tobacco and alcohol use [23]. Adolescents were classified in one of three following groups according to behavioral aggregation: healthiest (cluster 1), intermediate (cluster 2) and least healthy (cluster 3).

**Figure 1:**
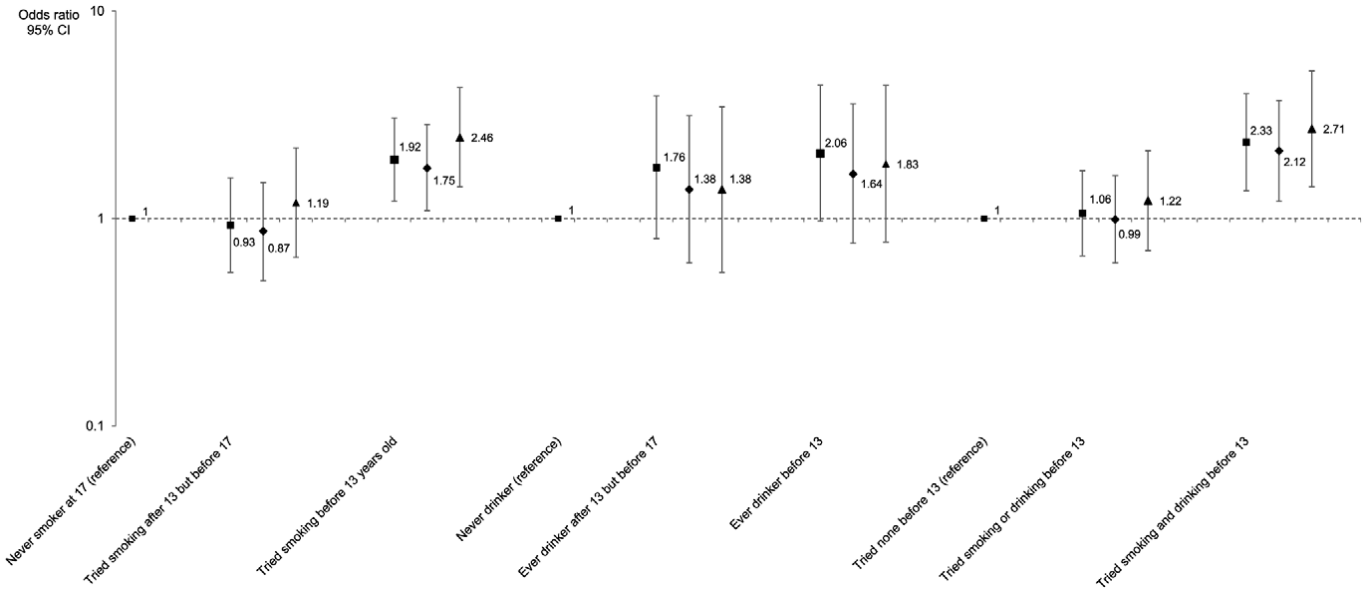
Associations between smoking and drinking and low forearm bone mineral density in late adolescence. Odds ratios and 95% confidence intervals for low bone density (z-score below −1 at 17 years of age) are presented adjusted for menarche age and regular sports practice at 17 years of age (squares), and with additional adjustment for body mass index at 17 (diamonds) or for bone mineral density at 13 years of age (triangles).

Figure 1 presents crude and adjusted odds ratios and 95% confidence intervals for the associations between smoking and drinking precocity and BMD z-score category. When considered dichotomously, there were clear significant associations between low 17 years BMD (z-score<−1) and having ever smoked by 13 years of age (OR = 1.92; 95% CI: 1.21, 3.05, adjusted for menarche age and sports practice) as well as with ever smoking and drinking in the same period (adjusted OR = 2.33; 95% CI: 1.36, 4.00). Associations remained significant after adjustment for body size or for baseline BMD. Although with lower precision and no statistical significance, an increasing trend of lower BMD z-score was found with increasing drinking precocity. Similarly to the results obtained with the linear model, there was an absence of dose-response relations between smoking and/or drinking frequency and BMD z-score categories.

## Discussion

In the present study, early initiation of smoking and alcohol drinking was inversely associated with late adolescence forearm bone mineral density in girls. This suggests a long-term association of these behaviors with bone accrual. These relations were not apparent when we quantified associations between smoking and drinking and bone mineral density at the same age. In the age range of our study, comparatively short exposure periods and cumulative doses may explain the absence of short-term associations and they may also be accountable for the observed lack of any significant dose-response relations between the reported frequency of smoking or drinking and bone quality.

Mean BMD estimates were adjusted for menarche age and sports practice to overcome the confounding effect of these factors, since they are not only associated with smoking and drinking but also determinants of bone quality. We also tested the possibility of confounding by several other factors (height, nutrient intake, supplement use, oral contraception, and parental education) which were not adjusted for in the final analysis, since they were not simultaneously associated with bone mineral density and smoking or drinking. We found lower average BMI as well as lower fat-free mass in late adolescence among girls who had experienced smoking and/or drinking by 13 years of age, suggesting that body size may be a relevant intermediate step in possible effects of these behaviors on bone quality. In agreement, we observed an attenuation of the associations between smoking or drinking and BMD after body size adjustment. This is to be expected if BMI is a mediator, since adjustment for body size should substantially decrease the component of the total effect operating through that path, resulting in weaker overall associations.

Previous research pertaining to this life stage is scarce but results are overall consistent with our findings. In a small prospective study of 9th grade adolescents, smoking and drinking experience were negatively associated with 12th grade bone mineral density [15]. Additionally, a 2-year follow-up study in a small sample of female University students showed an unfavorable development of bone mineral density in young women smokers who did not use oral contraceptives [18]. In a trial of an oral contraceptive, a higher mean loss of BMD was found among female adolescents who used alcohol [16]. However, another larger cross-sectional study in young women failed to find associations between smoking or drinking and forearm bone mineral density [13]. Nevertheless, a study in young men found a negative association of smoking with bone mineral density and cortical thickness [12]. More recently, a cross-sectional study reinforced this hypothesis, since smoking was associated with higher frequency of previous fracture and with worse bone properties in young male siblings. These associations were stronger in men whose smoking initiation occurred at an early age, suggesting possible impairment of optimal peak bone mass and geometry [26]. Our results are in agreement with this negative influence of smoking and drinking on bone health, particularly regarding the potential role of precocity, but extend it, by providing evidence of long-term associations between smoking and alcohol intake and bone quality in a large sample of girls selected from the general population.

While there is little research before peak bone mass accrual, an association between smoking and the amount of skeletal mass has long been described in adult twins, in whom it was estimated that smoking one pack of cigarettes daily throughout adulthood would lead to a 5 to 10% deficit in bone density by menopause [27]. Smoking has been hypothesized to decrease bone mineral density by interfering with body weight and fat mass, respectively by decreasing mechanical loading on the skeleton and by diminishing estrogen synthesis and leptin secretion by the adipose tissue [9]. Smoking may also act on bone metabolism by promoting accelerated estrogen metabolism and elimination, decreasing serum levels of calciotropic hormones, and increasing the secretion of adrenocortical hormones [28].

A recent systematic literature review showed that adult men and women reporting moderate alcohol intake had higher bone mineral density than abstainers [29]. Whereas direct and hormone-mediated mechanisms have been proposed to account for this protective effect of ethanol, inhibition of bone resorption seems to be the most likely mechanism involved in this relation [30], [31]. Despite a possible protective effect of moderate consumption, studies in clinical samples of heavy drinkers suggested excessive alcohol intake as a risk factor for lower bone mass [32]. This is supported by animal models, where impairing formation through an action on osteoblast activity appears as the most relevant mechanism by which alcohol interferes with bone health [10], [32]. This finding has particular importance during the first decades of life, since the balance between bone formation and resorption favors formation, resulting in a cumulative increase in bone mass, as well as in changes in microarchitecture and geometry, with consequent contribution to overall bone physical properties [1]. Therefore, the interference of ethanol on bone formation may be especially relevant during that life stage. An additional issue with growing public health relevance from young ages is the possible interference of binge drinking with bone accrual. Although evidence from studies in human populations remains scarce, a recent review has identified interesting results from animal models where binge drinking was found to have short- and long-term deleterious effects on the teenage rat skeleton, through increased resorption and decreased formation [33]. In our study adolescents were not specifically questioned about binge drinking, but we found weak, non-significant associations between the history of excessive alcohol consumption at 17 years of age (ever having felt drunk, age at the first drunkenness episode, and cumulative number of drunkenness experiences throughout life) and bone mineral density (results not shown). However, the significance of this lack of association is unclear, since exposure assessment in our study was not designed to explore the effect of binge drinking.

It should be noted that although there is a number of plausible biological mechanisms by which smoking and alcohol may affect bone quality, epidemiological evidence of the association between these exposures and bone mineral density remains inconsistent in adult women [34]. In fact, there is substantial indication that smoking and drinking tend to cluster with a number of other unhealthy behaviors which may themselves have a relevant effect on bone health [23]. One possible explanation for the associations found in the present study is indeed that these behaviors are not causal exposures but markers of other adverse influences, such as poor nutrition or sedentarism. Nevertheless, we observed that the associations between bone density and behavioral clusters (which included smoking, drinking, physical activity, fruit intake and sleeping hours) were weaker than those estimated between bone density and smoking or drinking alone. Although this does not exclude the possibility that smoking and drinking are risk markers instead of risk factors, it suggests that the other behaviors included in the cluster definition are not the main causal influences responsible for the observed associations.

### Limitations

In all prospective studies, differential losses to follow-up and non-random missing data may be important threats to validity since they may yield biased estimates. In the present study, girls who were excluded from the analysis because of missing data or losses to follow-up reported lower frequency of alcohol drinking and simultaneously had higher bone mineral density at 13 years of age. This is in agreement with our findings of an inverse association between this behavior and bone properties among girls included in the present analysis. Although this consistency does not clarify the extent of possible bias, it suggests that the direction of our results was probably not affected by differential information losses.

A limitation of the present work is that we used forearm BMD to summarize bone properties, and peripheral measures of areal bone mineral density obtained by dual-energy X-ray absorptiometry are not perfect substitutes for axial or total body measures, since they are affected by bone size [35]. Whole-body DXA would have provided a more accurate estimate of the systemic effects under study, as well as allowing for the estimation of bone mineral content variation, which may be a more informative parameter in studies conducted during growth [36]. However, the large sample size and community-based setting of evaluations required a practical and portable method for bone quality assessment. An additional aspect is that areal bone mineral density in children can fail to capture true volumetric density since it partly reflects bone size in addition to density and complementary data on other physical properties or geometry, such as bone area or mineral content, were not available. However, it should be noted that the mechanical resistance of bone to trauma is a function of several properties of bone tissue, including size [37]. Therefore, areal bone mineral density may be seen as a combined result of two important bone properties that determine strength: density and size. Even though this view is arguable, its rationale is supported by prospective evidence that areal bone mineral density is a good marker of fracture risk, as observed in a systematic review of studies in children [38].

In our study, pubertal development status was ascertained using menarche age. We acknowledge that menarche occurs relatively late in the pubertal development process and is not a perfect substitute for physical examination [39]. However, it becomes particularly useful in population studies of large samples where the feasibility of physical examination is limited. Nevertheless, menarche age is believed to be reproducible in the short term, as shown in a Canadian study of adolescent girls, where over three quarters of participants were able to recall menarche age within 1 month [40]. Regarding physical activity, even though the questionnaire applied is widely used by physical activity and exercise experts in Portugal, it did not undergo a formal validation procedure against accelerometer measurements. Nevertheless, the questionnaire was previously found to have high reproducibility [22].

An additional limitation of our study pertains to the possibility that the self-report of adverse health behaviors such as smoking and drinking by adolescents may be subject to misreporting due to both cognitive factors, such as comprehension and recall, and to situational influences, namely social desirability and interviewing conditions [41]. Moreover, the relatively low frequency of regular smoking and drinking decreased the statistical power for the analysis of potential dose-response relations between these behaviors and bone mineral density, which could reinforce the plausibility of our findings. Another limitation is the fact that we have no record about accumulated exposure to tobacco or alcohol between evaluations. In order to address this, we used precocity in the initiation of those behaviors as a proxy for the duration of exposure.

Despite the above-mentioned constraints, we were able to quantify the associations under study using a prospective design and during a period that spanned most of adolescence. Importantly, girls in this sample were born in the same year, which minimized confounding by cohort or period effects. We were also able to test a substantial number of other potential confounders of the effect under study.

In the present prospective study conducted in community adolescent girls, early initiation of smoking and alcohol drinking were associated with lower forearm bone mineral density in late adolescence. These behaviors may be relevant red-flags for impaired long-term bone mineral acquisition up to peak bone mass.
